# Role and impact of telemedicine in spine surgery: a systematic review

**DOI:** 10.1530/EOR-2025-0020

**Published:** 2025-12-05

**Authors:** Hussayn Shinwari, Abith Ganesh Kamath, Saran Singh Gill, Kapil Sugand

**Affiliations:** ^1^ Faculty of Medicine, St George’s University of London, London, United Kingdom; ^2^ Faculty of Medicine, Imperial College London, London, United Kingdom

**Keywords:** telemedicine, telehealth, spine surgery, remote consultation, postoperative care, patient outcomes, rehabilitation, digital health

## Abstract

**Purpose:**

- Telemedicine has emerged as a valuable tool in spinal surgery, enabling remote consultations, preoperative assessments, and postoperative care. This systematic review evaluates its impact on clinical outcomes, patient satisfaction, and healthcare resource use from 2020 to 2024, highlighting key benefits, challenges, and future research directions.

**Methods:**

- A systematic search of PubMed/MedLine, Scopus, Web of Science, and Embase identified studies assessing telemedicine in patients undergoing spinal surgery. Covidence (Covidence, Melbourne, Australia) was used for screening and selection. Risk of bias was evaluated, and a qualitative synthesis was conducted following SWiM guidelines due to study heterogeneity. Data were standardised to one decimal place, except for reported *P*-values.

**Results:**

- In total, 37 studies comprising 91,139 patients met inclusion criteria. Telemedicine demonstrated high diagnostic accuracy, with concordance rates exceeding 90% in most studies. Patient satisfaction remained consistently high (86.9–96.4%). It improved treatment adherence, pain management, and functional outcomes. Key advantages included cost-effectiveness and increased accessibility, though challenges such as bias and technological limitations persist.

**Conclusion:**

- Telemedicine plays a significant role in spinal surgery, offering high diagnostic accuracy, patient satisfaction, and improved clinical outcomes, including reduced pain and disability. It enhances compliance, reduces costs, and improves access to care. However, technological barriers, regulatory concerns, and study heterogeneity underscore the need for further research. Telemedicine holds promise for transforming spinal surgery care.

## Introduction

### The rise of telemedicine in spinal surgery: a pandemic-driven evolution

Telemedicine has emerged as a transformative tool in healthcare delivery, enabling remote consultations, preoperative assessments, postoperative monitoring, and continuity of care (1, 2, 3, 4). This evolution has been particularly impactful in spinal surgery, a field where accessibility, ongoing care, and monitoring are vital for optimising clinical and patient outcomes. The unprecedented global COVID-19 pandemic acted as a catalyst for the widespread adoption of telemedicine, driving innovation and accelerating its integration into routine clinical practice. This is due to various factors, including social distancing requirements, healthcare system strain, and increased innovation and investment in the platform (5). However, since then, telemedicine has been widely adopted into post-pandemic care.

### Benefits of telemedicine: convenience, efficiency, and accessibility

The use of telemedicine has broadened, driving rapid advancements in digital health technologies such as artificial intelligence, wearable devices, and improved communication platforms designed for remote care (6). Many patients preferred this avenue of treatment during the COVID-19 pandemic, with 88% of patients agreeing that virtual consultation was more convenient for them than an in-person visit (7). Telemedicine also offers several recognised benefits, including improved time efficiency, cost savings for both patients and healthcare providers, and enhanced community access to care (8).

### Advancing insights: the role of technology and limitations of pre-pandemic reviews

Healthcare surveillance opens the door for clinicians to obtain better insight into their patients through digital health tracking technologies and services (9). Telemedicine services have already been associated with improved healthcare outcomes while remaining on telemedicine in spinal surgery contained studies conducted before the onset of the pandemic; however, the scope of its findings was limited to an era when telemedicine adoption was not yet pervasive (2).

### Aims and objectives

This systematic review aimed to evaluate the literature on telemedicine in spinal surgery published between 2020 and 2024. By examining evidence from this pivotal timeframe, the review sought to assess the impact of telemedicine on clinical and patient-reported outcomes, patient satisfaction, healthcare resource utilisation, and barriers to its implementation.

## Methods

### Definition

Telemedicine was defined as any form of intervention or communication via online, digital, or phone-based platforms between spine surgery patients and their surgeons or clinical staff, as defined by Kolcun *et al.* (10).

### Adherence to guidelines

**Figure 1:**
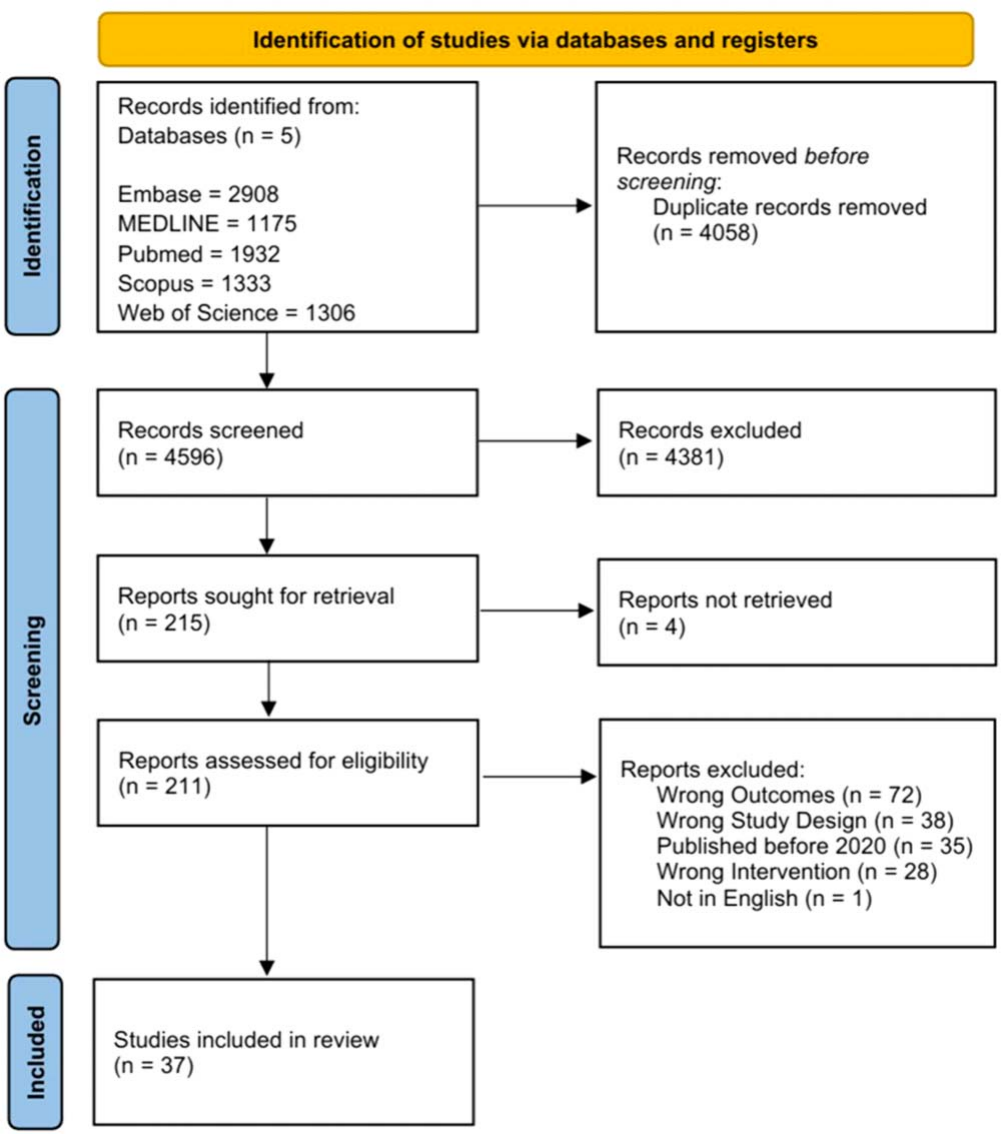
PRISMA 2020 flow diagram for new systematic reviews, which included searches of databases and registers only.

The systematic review was conducted in line with the Preferred Reporting Items for Systematic Reviews and Meta-Analyses (PRISMA) 2020 guidelines (11). The completed PRISMA flowchart is shown in Fig. 1. The study was registered in the PROSPERO Database (CRD42025626004).

### Search strategy

The literature search was carried out on the 7th of September 2024. PubMed/MedLine, Scopus, Web of Science, OVID, and Embase were searched. Search strings using MeSH terms are listed in Table 1. The inclusion and exclusion criteria are outlined in Table 2.

**Table 1:** Search string.

Search String
(Telemedicine OR telehealth OR telesurgery OR ((remote OR virtual) AND surgery)) AND (spinal OR spine OR spin*) AND (surgery OR surgical OR surg*) AND (outcomes OR procedures OR technologies OR remote OR robotic*)

**Table 2:** Eligibility criteria.

Inclusion	Exclusion
Studies using telemedicine-based interventions on patients with spine or spinal cord pathology that would require surgical care	Non-English publications
Studies published from 2020 onwards	Studies published before year 2020
Randomized controlled trials (RCTs), cohort studies, case–control studies, case reports, and database analyses	Reviews, editorials, conference abstracts, books, and consensus statements
Adult and paediatric patients	Grey literature
Studies that provide outcome data on telemedicine-based approaches	Non-peer-reviewed journal publications

#### Screening

The initial abstract screening was conducted by three reviewers using Covidence software (Covidence,  Australia). The first round consisted of screening titles, followed by abstracts, and then full texts for the final analysis. Any disagreements during the selection process were resolved through discussion, with final arbitration by the fourth reviewer when necessary.

#### Data extraction

Relevant data were extracted and collated on a spreadsheet using Microsoft Excel (Microsoft, USA). Collected information encompassed study characteristics, including title, authors, country of publication, publication date, outcomes assessed, conclusions, and sample sizes. In cases where data were missing, corresponding authors were contacted. Supplementary Table 1 (see section on Supplementary materials given at the end of the article) summarises key findings. Supplementary Table 2 summarises key clinical and functional outcomes.

### Risk of bias

**Figure 2:**
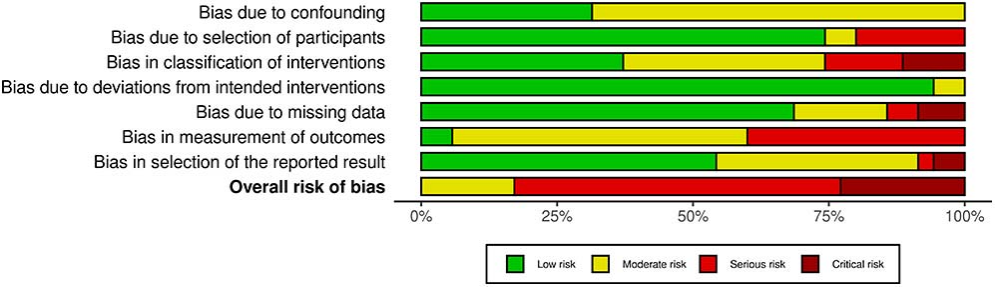
Risk of bias analysis (35 studies) using the risk of bias in non-randomised studies of interventions (ROBINS-I) tool.

**Figure 3:**
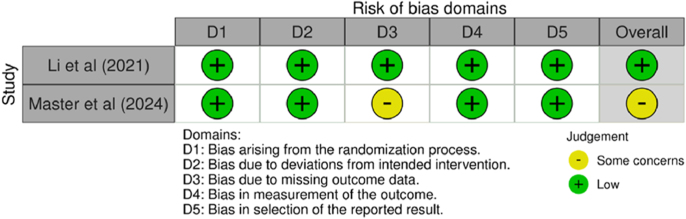
Risk of bias analysis (2 studies) using the Cochrane RoB 2 tool for randomised trials.

Two independent reviewers assessed the risk of bias for all included studies using the RoB 2 tool for randomised controlled trials and the ROBINS-I tool for non-randomised studies (10, 11), as seen in Figs 2 and 3. Discrepancies between reviewers were resolved through discussion, and, if necessary, a final decision was made by a third reviewer. The risk of bias assessment focused on key domains such as randomisation, blinding, and handling of missing data, ensuring the validity and reliability of the results.

#### Data analysis, qualitative synthesis, and reporting

Due to methodological heterogeneity, including variations in study design, patient populations, interventions, and reported outcomes, a meta-analysis was not feasible. Instead, following the synthesis without meta-analysis (SWiM) guidelines, a qualitative synthesis was conducted (12).

## Results

### Study selection

A total of 8,654 articles were initially identified through comprehensive database searches of PubMed/MedLine, Scopus, Embase, and Web of Science (Fig. 1). After removal of duplicates and detailed screening against predefined inclusion and exclusion criteria (Table 2), 37 studies involving a total of 91,139 patients were deemed eligible and included in the final analysis (Supplementary Table 1) (13, 14, 15, 16, 17, 18, 19, 20, 21, 22, 23, 24, 25, 26, 27, 28, 29, 30, 31, 32, 33, 34, 35, 36, 37, 38, 39, 40, 41, 42, 43, 44, 45, 46, 47, 48).

### Risk of bias

Among the 35 non-randomised studies assessed using the ROBINS-I tool, risk of bias varied across domains (13, 14, 15, 16, 17, 18, 19, 20, 21, 23, 24, 25, 26, 27, 28, 30, 31, 32, 33, 34, 35, 36, 37, 38, 39, 40, 41, 42, 43, 44, 45, 46, 47, 48). Confounding was low in 28.6% of studies and moderate in 71.4%. Selection of participants was predominantly low risk (74.3%), with smaller proportions classified as serious (20.0%) or moderate (5.7%). Classification of interventions was evenly split between low and moderate risk (37.1% each), with 14.3% serious and 11.4% critical. Deviations from intended interventions were largely low risk (94.3%). Missing data posed greater challenges: 68.6% low, 17.1% moderate, and 14.3% serious or critical combined. Measurement of outcomes was moderate in 54.3%, serious in 40.0%, and low in 5.7% of studies. Selection of reported results was predominantly low risk (54.3%), with moderate (37.1%), serious (2.9%), and critical (5.7%) studies making up the remainder. Overall, 60.0% of studies carried a serious risk of bias, 22.9% critical, and 17.1% moderate.

Two RCTs were assessed using the RoB 2 tool. Li *et al.* demonstrated low risk of bias across all domains, resulting in an overall low risk, whereas Master *et al.* had some concern due to missing outcome data, with all other domains judged low risk, resulting in an overall assessment of some concern (22, 29).

### Diagnostic consistency

Thirteen studies demonstrated high diagnostic consistency for telemedicine in spine surgery, often matching or exceeding face-to-face (F2F) consultations. Sharma *et al.* and Rappard *et al.* reported 100% agreement rates in interventional and surgical plans, while Jansen *et al.* observed 94% (13, 18, 25). Some of the studies reported a ‘diagnostic consistency’ metric, where Hobson *et al.* found 79% of telemedicine consultations achieved diagnostic consistency exceeding 75%, and Melian *et al.* reported only 0.5% diagnostic errors (23, 36). In addition, Prasse *et al.* confirmed strong agreement in pain location and neurophysiological assessments, with kappa values ≥0.7 (26). Another example is Ye *et al.*, which noted similar diagnostic accuracy between telemedicine (79.5%) and F2F methods (82.6%), with no significant differences (*P* = 0.7) (17).

### Patient satisfaction with telemedicine

Patient satisfaction with telemedicine was consistently high across studies. Satin *et al.* reported 87.7% satisfaction, with 70% rating their experience as ‘very satisfied’ (28). Melian *et al.* found a mean satisfaction score of 9.9/10, and Bombardier *et al.* reported 91% satisfaction (36, 39). As one of the highest, Zhu *et al.* noted a 96.4% satisfaction rate, and Hobson *et al.* (23) reported 93% satisfaction in the technical domain (20). Most patients (80%) expressed a preference for telemedicine for future care. There is also widespread acceptance of the platform, where Goyal *et al.* found that 86.9% of patients would recommend telemedicine, further reinforcing its acceptance and value in spine care (48).

### Patient-reported outcome measures (PROMs)

Telemedicine interventions significantly improved PROMs compliance and outcomes. Some of the studies represented enhanced adherence, where Leyendecker *et al.* and Farias *et al.* observed compliance rates as high as 94%, aided by tools such as the SPINEhealthie app (19, 34, 41). The SPINEhealthie app facilitated significant gains in functionality and pain management, offering a convenient tool for patient recovery at home (24). SPINEhealthie is an app that helps patients recover after spine surgery. It lets them track their progress, learn about spine health, and stay connected with their doctors from home.

Similarly, Bovonratwet *et al.* found similar PROMs adherence rates between telemedicine and in-person groups (31). Other studies also reported improved mental health, where Woznica *et al.* reported a 3.4-point improvement in PROMIS-MH scores (*P* = 0.03), emphasising the mental health benefits of telemedicine (45).

### Clinical outcome measures

Telemedicine was effective in reducing pain and improving functionality: Han *et al.* observed reductions in visual analogue scale (VAS) scores from 8.2 ± 0.1, and Perna *et al.* noted decreases from 7.6 ± 1.7 to 3.4 ± 1.2 within 15 days (16, 32). There was also improved functionality, with Leyendecker *et al.* highlighting substantial improvements in neck and back pain (41), and Rappard *et al.* reported significant Oswestry Disability Index (ODI) reductions (e.g. from 52, 95% CI: 50–54) (18).

### Effect of patient age on telemedicine outcomes

The effectiveness of telemedicine was consistent across diverse age groups. Studies included a wide range of participants, from paediatric cases averaging 15 ± 3.7 years to older cohorts with a mean age of 68.9 ± 2.5 years (19, 33). There were also quality-of-life improvements, where Woznica *et al.* documented significant enhancements in pain relief (91.6%, *P* < 0.0001) and health-related quality of life (45). These findings reinforce the versatility and reliability of telemedicine across various demographics and clinical contexts.

## Thematic content analysis

### Spine care and management

Telemedicine has provided significant benefits for spine care, improving patient satisfaction and clinical outcomes. Lightsey *et al.* reported that patients highly preferred telemedicine follow-ups for spine care due to convenience and accessibility, with satisfaction scores based on Likert scales ≥3 out of 5 (35). Perna *et al.* demonstrated that combining telemedicine with semirigid corset therapy for acute low back pain reduced VAS scores from 7.6 ± 1.7 to 3.4 ± 1.5 (55.2%) within 15 days, achieving better outcomes compared to standard care (32). Woznica *et al.* observed significant mental health improvements, with PROMIS-MH scores increasing by 3.4 points (*P* = 0.03) among patients managed via interdisciplinary telemedicine for low back pain (45). Shafi *et al.* highlighted high patient satisfaction with telehealth, reporting mean satisfaction scores of 4.8/5 (±0.5) (49). For chronic spinal cord injury-related pain, Bombardier *et al.* demonstrated that telehealth-delivered hypnotic cognitive therapy reduced pain intensity by 1.28 points from 5.4/10 (23.7%), with 91% of participants reporting that they were satisfied (39).

### Spine surgery and operative interventions

Telemedicine has shown efficacy in supporting preoperative and postoperative spine surgery care. Greven *et al.* reported a diagnostic consistency of 91% for virtual preoperative assessments, showing equivalence with in-person evaluations (37). Smartphone-based remote monitoring, as shown by Leyendecker *et al.*, improved compliance by 45% and significantly reduced VAS scores for neck and back pain at the 90-day follow-up (41). Balu *et al.* highlighted high satisfaction levels with remote therapeutic monitoring, achieving a mean Likert score of 4.2/5 and adherence rates of 94% (42). Totala *et al.* observed that telemedicine follow-ups for spine surgery achieved an overall success rate of 82.9%, with only a fifth of patients requiring in-person visits (43). In addition, Tian *et al.* demonstrated that 5G-enabled telerobotic spinal surgery provided safe and accurate outcomes (46).

### Outpatient and ambulatory spine care

Outpatient spine care has benefited significantly from telemedicine, enhancing accessibility and reducing logistical burdens. Greven *et al.* reported a 95% satisfaction rate for telemedicine consultations in patients, with many preferring telemedicine due to reduced travel and associated costs (37). Early postoperative care also benefited, as Master *et al.* observed a 21% improvement in adherence to physical activity plans, with ODI scores improving by 26 points at 3 months (29). Similarly, Prasse *et al.* demonstrated that remote monitoring following full-endoscopic spine surgery significantly increased compliance over time and improved patient-reported outcomes (26).

### Specialised spinal and orthopaedic conditions

Telemedicine has been transformative for managing specialised spinal conditions. Melian *et al.* reported that 87.5% of patients preferred teleconsultations for postoperative visits, achieving an overall satisfaction rate of 99.5% (36). Shafi *et al.* highlighted that 81% of patients reported being ‘extremely satisfied’ with telemedicine, matching in-person satisfaction levels (49). For traumatic spinal cord injuries, Li *et al.* found that SF-36 scores improved at the 6-month follow-up, although there were no significant differences at discharge (22). In adolescent idiopathic scoliosis, Zhu *et al.* observed a 96.4% satisfaction rate with real-time compliance monitoring, which significantly improved adherence to treatment over the study period (20).

## Discussion

### Principal findings

This systematic review aimed to evaluate the role of telemedicine in spine surgery, focusing on key aspects such as patient satisfaction, diagnostic accuracy, cost savings, and treatment compliance. Our findings provide compelling evidence that telemedicine is a feasible and reliable alternative to in-person care, offering comparable outcomes for both pre- and postoperative management. These results align with the broader integration of telemedicine into surgical practices, aimed at improving healthcare accessibility, enhancing efficiency, and optimising resource utilisation. However, despite these promising outcomes, challenges such as variability in physical examination techniques, limited technological access, and regulatory barriers remain, highlighting the need for continued innovation and further research in this field.

### Patient satisfaction and diagnostic accuracy

A critical measure of effectiveness in telemedicine is patient satisfaction, and our systematic review found a strong satisfaction rate of 86.5%. This aligns with other research indicating even higher satisfaction levels, such as the 97.6% satisfaction reported by Bhuva *et al.* (50). This high level of satisfaction underscores the potential to meet patients’ needs and expectations via telemedicine, especially in the context of spine surgery, where frequent follow-ups and consultations are necessary.

In terms of diagnostic accuracy, our systematic review revealed that only 4% of cases saw changes in surgical plans following telemedicine consultations, primarily due to new imaging findings or an increase in the number of vertebral levels operated on. This is consistent with findings from previous studies, such as Ye *et al.*, which reported no significant difference in the accuracy of surgical plans between telemedicine and in-person evaluations (79.5 vs 82.6%; *P* = 0.7) (44). Notably, modifications to surgical plans were predominantly related to an additional level to the operation, with changes in approach or procedure occurring less frequently. These results support the reliability of telemedicine for surgical planning, with minimal adjustments required compared to in-person consultations.

### Pain management and rehabilitation capabilities

This review has confirmed the efficacy of telemedicine in managing spinal pain and contributing both to clinical and patient-related outcomes. In the management of low back pain, telerehabilitation (TR) has been shown to be as, if not more, efficient than in-person care, with a trend towards higher effectiveness when all visits were conducted via TR (51). This supports the notion that telemedicine, particularly in the form of telerehabilitation, can be a highly effective tool in managing spinal conditions, offering both convenience and high-quality care.

There were significant improvements in pain and disability scores for patients using telemedicine, with reductions in VAS scores from 8.2 to 2.2 (73.2% decrease) and ODI scores from 52 to 35 (32.7% decrease). These results are consistent with those observed by Gialanella *et al.*, who reported greater declines in neck pain and disability for patients in home-based telemedicine groups compared to controls (*P* < 0.001) (52). These improvements emphasise the potential of telemedicine to not only maintain but enhance the quality of care for spine surgery patients, particularly in terms of pain management and functional recovery.

### Cost savings and efficiency

In addition to clinical outcomes, this review identified significant cost savings associated with telemedicine. These savings include an estimated $793,835 in hospital costs, reduced missed appointments, and faster postoperative care (17, 21, 34). Patients also benefited from substantial time savings, avoiding long wait times and the need for travel to healthcare facilities. This is consistent with cost analyses that show telemedicine can save patients an average of 4.1–5.6 h per visit, resulting in financial savings of up to $223.3 ± 171.4 per visit (53). Furthermore, a U.S. telehealth model that replaced home visits doubled nurses’ caseload capacity and saved 43,560 driving minutes over 14 months (54), while a Canadian study reported annual savings of Can $65,520 (US $49,584.14) by replacing in-person visits with telehealth (55). These findings further underscore the efficiency and cost-effectiveness of telemedicine, benefiting healthcare systems, resource allocation, and patients.

### Improved compliance and accessibility

Moreover, our review found a significant increase in treatment plan compliance, with adherence rising from 49.1 to 80.5% (*P* < 0.05). Follow-up rates were also high, with 92% of patients following up on physical activity and 95% on patient-reported outcomes (PROs) at 6 months (56). Telemedicine not only improved patient satisfaction and clinical outcomes but also enhanced patient engagement, autonomy, and compliance with management plans.

The integration of telemedicine into clinical practice, particularly in spine surgery, holds immense potential for revolutionising healthcare delivery. It offers increased accessibility, reduced costs, and improved patient outcomes, all of which are particularly valuable in underserved and rural populations. However, to fully realise this potential, it is essential to address challenges such as limited technological access, regulatory barriers, and reimbursement issues (57). For telemedicine to be sustainable in spine surgery, clear billing codes, improved internet access, and enhanced healthcare provider training in telemedicine are crucial.

### Ethical considerations

Telemedicine in spine surgery raises important ethical issues, particularly regarding patient confidentiality, data protection, and informed consent when consultations occur on virtual platforms (58). Equity of access also remains a concern, as patients from lower socioeconomic backgrounds or rural regions may face barriers to reliable technology, potentially widening healthcare disparities (59). Ensuring that telemedicine enhances rather than compromises patient safety and autonomy is therefore essential.

### Limitations

The heterogeneity of included studies, such as variations in study designs, telemedicine platforms, and patient populations, made direct comparisons challenging. In addition, another limitation was the predominance of data from more economically developed countries, which may not fully represent the challenges or opportunities in low-resource settings, where telemedicine could have the most significant impact due to poor accessibility or a substantially high clinician-to-patient ratio. Furthermore, a critical limitation was the high risk of bias in the majority of the papers included in the study, which undermines the reliability of the findings.

### Future research

Future research should focus on improving the accuracy of virtual physical examinations, particularly when assessing neurological deficits, provocative testing, and identifying myelopathy (60). Investigating the legal and regulatory challenges of providing telemedicine services across borders, such as licensure, malpractice, and liability issues, is also essential to ensure safe and effective care delivery (61). Training and certification requirements for healthcare providers using telemedicine in spine surgery ought to be defined to ensure that consultations and care meet the highest standards of quality for prioritising patient safety (49).

## Conclusion

Telemedicine has emerged as a valuable adjunct to in-person consultations in spine surgery, demonstrating comparable diagnostic accuracy, improved patient outcomes, and meaningful cost savings, while enhancing accessibility and engagement, particularly for underserved populations. Its integration, however, must be guided by ethical and practical considerations, including safeguarding patient confidentiality, ensuring equitable access, and preserving the patient–clinician relationship, alongside addressing technological and regulatory barriers. When implemented responsibly, telemedicine has the potential to transform spine surgery, delivering care that is not only effective but also accessible, ethical, and sustainable in the digital era.

## Supplementary materials



## ICMJE Statement of Interest

The authors declare that there is no conflict of interest that could be perceived as prejudicing the impartiality of the work reported.

## Funding Statement

This work did not receive any specific grant from any funding agency in the public, commercial, or not-for-profit sector.

## Author contribution statement

HS and AGK were involved in conceptualisation, data curation, formal analysis, investigation, methodology, project administration, software, supervision, validation, visualisation, writing the original draft, and writing review and editing. SSG contributed to data curation, formal analysis, investigation, validation, and writing the original draft. KS was involved in conceptualisation, methodology, formal analysis, investigation, supervision, validation, visualisation, writing the original draft, and writing review and editing. All authors reviewed and approved the final manuscript.
