# Supplementary material for: Role and impact of telemedicine in spine surgery: a systematic review
Source: EFORT Open Rev. 2025 Dec 5;10(12):894–902. doi: 10.1530/EOR-2025-0020 (PMC12687113; doi:10.1530/EOR-2025-0020)
Supplement: Supplementary file 1 [file supplementary_materials.pdf]

Author	Title	Country	N	Intervention	Aim	Theme	Conclusion	Study Type
Sharma et al (2021)	The Utility of Remote Video Technology in Continuing Neurosurgical Care in the COVID-19 Era	USA	10772	Remote video technology (telemedicine)	To assess the impact of remote video technology on neurosurgical care and education during COVID-19.	Neurosurgical care and education	Remote video technology improved patient satisfaction and neurosurgical care delivery; it supported education and expanded care access.	Retrospective cohort study
Crawford et al (2022)	Telemedicine Visits Generate Accurate Surgical Plans Across Orthopaedic Subspecialties	USA	303	Telemedicine for surgical planning	To evaluate if surgical plans generated through telemedicine differ significantly from those made in-person.	Orthopaedic surgery across subspecialties	Telemedicine effectively generates surgical plans with 96% accuracy across orthopaedic subspecialties, suggesting high reliability.	Retrospective cohort study
Pennington et al (2022)	Positive Impact of the Pandemic: The Effect of Post-COVID-19 Virtual Visit Implementation on Departmental Efficiency and Patient Satisfaction in a Quaternary Care Center	USA	54562	Virtual neurosurgical consultations via telemedicine	To analyse the impact of telemedicine on workflow, care delivery efficiency, and patient satisfaction during the pandemic.	Neurosurgical outpatient care	Telemedicine maintained clinical volume, reduced appointment delays, and improved satisfaction without sacrificing efficiency.	Retrospective analysis of administrative data
Han et al (2021)	The Use of Remote Programming for Spinal Cord Stimulation for Patients With Chronic Pain During the COVID-19 Outbreak in China	China	64	Remote programming for spinal cord stimulation (SCS)	To investigate the demand and utility of remote follow-ups, including remote programming, for patients with chronic pain treated with SCS.	Chronic pain management	Remote programming was highly demanded and widely recognized for its efficiency, economic benefits, and time-saving advantages.	Observational study
Ye et al (2024)	Telemedicine Improves Access to Care for Spine Patients With Low Socioeconomic Status	USA	8197	Telemedicine for spine care	To compare missed appointment rates and evaluate socioeconomic status impacts on telemedicine and in-person appointments.	Spine care	Telemedicine significantly reduced missed appointments, particularly aiding patients with lower socioeconomic status, by overcoming traditional access barriers.	Retrospective cohort study
Rappard et al (2023)	Feasibility and Effectiveness of Telehealth in the Management of Cervicothoracic and Lumbar Pain During the First Six Months of the SARS-CoV-2 Pandemic	USA	101	Telehealth for spine pain management	To assess the effectiveness, utility, and feasibility of telehealth in managing spine pain.	Cervicothoracic and lumbar pain	Telehealth was feasible and effective, with outcomes comparable to pre-pandemic and non-telehealth spine care practices.	Retrospective case series
Leyendecker et al (2023)	Outpatient Fully Endoscopic Cervical Unilateral Laminotomy for Bilateral Decompression with Virtual Postoperative Monitoring	USA	23	Fully endoscopic cervical laminotomy with virtual monitoring	To evaluate the safety, efficacy, and impact of virtual postoperative monitoring in cervical laminotomy for bilateral decompression.	Cervical spinal stenosis	Endoscopic laminotomy with virtual follow-ups significantly reduced pain and hospitalizations while improving functional outcomes.	Retrospective cohort study
Zhu et al (2021)	A Compliance Real-Time Monitoring System for the Management of Brace Usage in Adolescent Idiopathic Scoliosis Patients: A Pilot Study	China	28	Real-time compliance monitoring system with a WeChat Mini Program	To evaluate the feasibility and effectiveness of a real-time monitoring system for brace treatment compliance in AIS patients.	Adolescent idiopathic scoliosis (AIS)	Real-time monitoring improved compliance and patient satisfaction with brace treatment, showing potential for large-scale application.	Pilot study
Cui et al (2021)	Effects of Telemedicine Triage on Efficiency and Cost-Effectiveness in Spinal Care	USA	10271	Electronic triage (E-triage) for spinal care	To evaluate cost savings and efficiency improvements with an E-triage system for spinal care.	Ambulatory spinal care	E-triage significantly reduced unnecessary visits, travel, and cost, improving efficiency and patient access to spinal care.	Retrospective review
Li et al (2021)	Effects of Online Home Nursing Care Model Application on Patients with Traumatic Spinal Cord Injury	China	80	Online home nursing care model	To evaluate the effectiveness of an online home nursing care model on the dysfunction index, quality of life, and complications of TSCI patients.	Traumatic spinal cord injury (TSCI)	Online home nursing reduced complication incidence, alleviated dysfunction, and improved quality of life for TSCI patients.	Randomized controlled trial
Hobson et al (2021)	A Multicenter Evaluation of the Feasibility, Patient/Provider Satisfaction, and Value of Virtual Spine Consultation During the COVID-19 Pandemic	USA	128	Virtual spine consultations	To assess feasibility, patient/provider satisfaction, and value of telehealth spine consultation during COVID-19.	Spine consultation	Virtual consultations were feasible with high patient/provider satisfaction; 91% had successful connection on the first attempt.	Multicenter observational study
Shah et al (2024)	Efficacy of Telerehabilitation for Spine Pain During the Coronavirus Pandemic Lockdown	India	428	Telerehabilitation and in-clinic multimodal rehabilitation	To compare the efficacy of telerehabilitation versus in-clinic rehabilitation for spine pain during COVID-19 lockdown.	Spine pain	Telerehabilitation significantly reduced pain and disability compared to in-clinic rehabilitation, indicating strong efficacy and potential for broader use.	Retrospective propensity score-matched analysis
Jansen et al (2021)	Spine Examination During COVID-19 Pandemic via Video Consultation	Germany	43	Video-based spine examination	To evaluate the feasibility of spine examination through video consultation compared to in-person methods during COVID-19.	Back pain	Video consultations reliably measured pain intensity, active range of motion, and basic neurophysiologic tests, though physical exams remain the gold standard.	Observational study
Prasse et al (2023)	Remote Patient Monitoring Following Full Endoscopic Spine Surgery: Feasibility and Patient Satisfaction	USA	71	Remote patient monitoring using SPINEhealthie app	To evaluate feasibility and satisfaction with postoperative monitoring using a mobile app after full endoscopic spine	Full endoscopic spine surgery	Remote monitoring was feasible and well-received, with significant engagement in app-based communication and PROM collection.	Prospective cohort study

					surgery.			
Meron et al. (2023)	Feasibility and Acceptability of a Telemedicine Triage Model Among Medicaid Patients	USA	33	Telemedicine triage	Evaluate feasibility and acceptability of telehealth for Medicaid patients	Low back pain	Feasible and acceptable; promising for those able to participate but limited by non-participation	Prospective cohort study
Satin et al. (2022)	Spine Patient Satisfaction with Telemedicine During the COVID-19 Pandemic	USA	772	Telemedicine consultations	Assess satisfaction with spine telemedicine during COVID-19	Spine-related issues	High satisfaction (87.7%); preference for telemedicine (45%); challenges with technical issues	Cross-sectional study
Prasse et al. (2023)	Remote Patient Monitoring Following Full Endoscopic Spine Surgery: Feasibility and Satisfaction	USA	71	Remote patient monitoring via mobile app	Investigate feasibility of remote monitoring post-surgery	Postoperative spine care	Feasible; high satisfaction with app (74.8% overall usability); improved compliance over time	Prospective observational study
Master et al. (2024)	Wearable Device and Telehealth Counseling Physical Activity Intervention	USA	16	Wearable device (Fitbit) + telehealth counselling	Examine feasibility and acceptability of wearables and telehealth counselling post-surgery	Early postoperative spine surgery	Feasible and acceptable; improved physical activity, MVPA, and no adverse events reported	Pilot randomized controlled trial
Bisson et al. (2021)	Association Between Patient Satisfaction and Mode of Visit in Orthopedics	USA	2049	Telemedicine vs. in-person orthopaedic visits	Examine patient satisfaction and predictors based on mode of care	Orthopaedic consultations	No significant satisfaction difference by mode of visit; satisfaction associated with race, provider relationship	Retrospective comparative study
Bovonratwet et al. (2022)	Telemedicine Visits Can Generate Highly Accurate Diagnoses and Surgical Plans for Spine Patients	USA	166	Telemedicine for preoperative spine assessment	Assess effectiveness of telemedicine in preoperative diagnosis and surgical planning	Spine surgery candidates	Telemedicine effectively establishes diagnoses; rare surgical plan changes (20%), mostly due to new imaging findings	Retrospective cohort study
Perna et al. (2021)	Importance of Telemedicine and Home Therapy During COVID-19 Pandemic: Role of Semirigid Corset in Acute Low Back Pain	Italy	127	Semirigid corset + telemedicine follow-up	Evaluate role of corset therapy in managing acute low back pain, minimizing in-person visits during COVID-19	Acute low back pain	Combined corset and drug therapy reduced pain and recovery time, increased satisfaction compared to drug therapy alone	Retrospective observational study
Sultan et al. (2020)	Utilization of Telemedicine Virtual Visits in Pediatric Spinal Deformity Patients	USA	189	Telemedicine virtual visits	Evaluate feasibility and patient satisfaction for telemedicine in paediatric spinal deformity patients	Paediatric spinal deformity	Telemedicine is feasible; shorter wait times and high satisfaction for surgeon performance and virtual visits	Retrospective cohort study
Farias et al. (2022)	Remote Patient Monitoring in Spine Surgery	Brazil	30	Remote monitoring platform using a smartphone app	Present experience of remote monitoring for post-operative spine surgery care	Lumbar spine surgery	Remote monitoring improves post-operative care, enhances quality of life, and detects complications early	Prospective observational study
Lightsey et al. (2021)	Patient Experiences of Telemedicine in Spine Care	USA	139	Telemedicine for spine care	Investigate patient satisfaction and preferences for telemedicine vs. in-person care	Spine-related care	High satisfaction for telemedicine, preference for in-person first visits, telemedicine favoured for follow-ups due to convenience	Mixed methods study
Melian et al. (2021)	Teleconsultation in the Management of Elective Orthopedic and Spinal Conditions During COVID-19 Pandemic	New Zealand	853	Teleconsultation vs. in-person consultations	Evaluate preference, satisfaction, and consultation duration for teleconsultation	Elective orthopaedic and spinal procedures	Teleconsultation preferred for follow-ups, shorter consultation times, high satisfaction; role suggested beyond COVID-19	Prospective cohort study
Greven et al. (2021)	Telemedicine in the Evaluation and Management of Neurosurgical Spine Patients	USA	346	Telemedicine consultations	Compare telemedicine satisfaction to in-person visits; assess feasibility for surgeries and procedures	Spinal pathology	High satisfaction with telemedicine; reduced time and socioeconomic burden; supports telemedicine for routine and surgical evaluation.	Retrospective cohort study
Shafi et al. (2020)	The Efficacy of Telehealth for the Treatment of Spinal Disorders	USA	84	Telehealth videoconferencing	Evaluate patient satisfaction with telehealth for spinal disorders	Spinal pathology	High satisfaction with telehealth; effective for communication and treatment planning, though limited for physical exams.	Prospective observational study
Bombardier et al. (2024)	The Efficacy, Safety, and Satisfaction of Telehealth-Delivered Hypnotic Cognitive Therapy for Chronic Pain in Spinal Cord Injury	USA	44	Telehealth-delivered hypnotic cognitive therapy (HYP-CT)	Evaluate effectiveness of telehealth-delivered HYP-CT for chronic pain in SCI compared to historical in-person controls	Chronic SCI-related pain	Telehealth HYP-CT reduced pain intensity and related measures, with effects comparable to in-person controls. Supports further randomized trials.	Pilot study with historical controls
Greven et al. (2022)	Telemedicine in Spine Surgery: Outcomes for 138 Patients With Virtual Preoperative Assessment Compared to Historical Controls	USA	276	Virtual preoperative assessment for spine surgery	Evaluate safety and efficacy of telemedicine for preoperative spine surgery evaluation	Spine surgery candidates	No significant difference in surgical outcomes, complications, or readmissions compared to in-person evaluations. Telemedicine is safe and effective.	Retrospective cohort study
Leyendecker et al. (2024)	Smartphone Applications for Remote Patient Monitoring Reduces Clinic Utilization After Full-Endoscopic Spine Surgery	USA	454	Smartphone app for remote monitoring (SPINEHealthie)	Assess impact of smartphone-based RPM on clinic visits and patient outcomes post-surgery	Full-endoscopic spine surgery	RPM via smartphone significantly reduced in-person follow-ups, increased follow-up compliance, and maintained patient safety. Promising for scaling.	Retrospective analysis
Balu et al. (2024)	Single-center Pilot Study of Remote Therapeutic Monitoring in Patients with Operative Spinal Pathologies	USA	21	Remote therapeutic monitoring with Fitbit	Investigate feasibility, satisfaction, and correlation of wearable device metrics with PROMs	Operative spinal pathologies	RTM showed high compliance and satisfaction, with moderate correlation between activity levels and PROMs. Highlights feasibility and potential for broader application.	Pilot study

Totala et al. (2024)	Telemedicine in Follow-up after Spine Surgery: Need of the Hour	India	84	Video teleconsultations	Evaluate role and success rate of telemedicine in spine surgery follow-ups; identify barriers to success	Spine surgery (including CVJ)	Telemedicine was 82.87% successful; more effective for follow-ups except for CVJ surgeries. Success correlated with socioeconomic and educational factors. Improvements in internet connectivity could enhance success rates.	Prospective observational study
Ye et al. (2022)	Similar Accuracy of Surgical Plans After Initial In-Person and Telemedicine Evaluation of Spine Patients	USA	131	Telemedicine preoperative assessments	Compare accuracy of surgical plans from telemedicine vs. in-person evaluations	Spine surgery candidates	Telemedicine evaluations produced surgical plans as accurate as in-person evaluations. Supports use for preoperative assessments in routine spine practice.	Prospective cohort study
Woznica et al. (2023)	Telemedical Interdisciplinary Care Team Evaluation and Treatment of People With Low Back Pain	USA	36	Telemedicine interdisciplinary care model	Evaluate impact of ICT on pain and health-related quality of life for patients with LBP	Low back pain (LBP)	ICT via telemedicine significantly improved pain and HRQoL. Low imaging, prescription, and interventional use rates. Promising for larger randomized studies.	Prospective pilot study
Tian et al. (2020)	Telerobotic Spinal Surgery Based on 5G Network: The First 12 Cases	Chin a	12	5G network-based telerobotic spinal surgery	Evaluate feasibility and accuracy of 5G telerobotic surgery for spinal disorders	Spinal disorders	5G telerobotic spinal surgery was accurate, safe, and reliable. Demonstrates great potential for rural areas with limited resources.	Case series
Low et al. (2024)	Outcomes and Patient Perspectives of a Novel Virtual Spinal Referral Pathway in a Non-Specialist Centre	Irela nd	44	Virtual multidisciplinar y team pathway	Evaluate efficiency and patient satisfaction with a novel virtual spine clinic pathway for referral management	Spinal disorders	Pathway was efficient in reducing wait times for consultations and interventions; high patient satisfaction reported.	Retrospective observational study
Goyal et al. (2020)	Development of a Telemedicine Neurological Examination for Spine Surgery	USA	41	Telemedicine neurological examination	Test feasibility and accuracy of telemedicine neurological examination for spine surgery	Spinal disorders	Comparable motor, sensory, and special test scores to in-person exams. High satisfaction rates; first validated telemedicine neurological exam for spine surgery.	Prospective validation study

Supplementary Table 1 – Study Characteristics

Author	Telemedicine followed by in person visit PROMs	VAS	ODI	Pain Regression	Patient Preferences	Age	Success	PROMS
**Balu et al (2024)**					3.64 at Day 30 (p=0.031) ; Willingness to use RTM again: 3.50 at Day 30 (not significant), 3.90 at Day 90 (p=0.031).; 3.70 at Day 90 (not significant). ; Patients reported willingness to continue RTM:	60+ years: 42.9%.; Age categories: ; 40–59 years: 42.9% ; <40 years: 14.3% ; Mean age: 56.7 years (SD: 15.3).	RTM demonstrated feasibility for tracking recovery and correlating activity levels with PROMs.	Positive correlation between changes in activity levels and PROMs, suggesting utility in monitoring functional recovery.
**Bisson et al (2021)**						Mean = 53.37 (SD = 18.13)		
**Bombardier et al (2024)**		Significance: Statistically and clinically significant reductions.; Pain intensity (likely on a 0–10 scale) reduced from 4.80 (baseline) to 3.52 (4 weeks) and 3.30 (12 weeks).		Pain reduction was significant in telemedicine: ; Effect size: −0.83 (4 weeks), −1.03 (12 weeks). ; 12 weeks: −1.50 points (31%). ; Significance: Clinically meaningful reductions in pain.; 4 weeks: −1.28 points (27%).		Mean age: 54.4 years (Z-HYP-CT group), comparable to historical controls (P = .869).	38% of participants achieved a ≥30% reduction in pain intensity at 12 weeks, indicating clinical success.	Pain catastrophizing: −5.04 at 12 weeks (P = .002). ; Pain intensity: −1.61 at 12 weeks (P < .0001). ; Depression severity: −2.21 at 12 weeks (P = .008). ; Pain interference: −1.00 at 12 weeks (P = .004). ; Pain intensity, pain interference, depression, and pain catastrophizing all showed significant improvements with Z-HYP-CT intervention. ; Significance: Robust and significant improvements in PROMs.
**Bovonratwet et al (2022)**	Preoperative ; SF-12 MCS: 51.9 ± 11.6 ;					53.6 ± 15.1 (Telemedicine only), 57.5 ±		SF-12 PCS, SF-12 MCS, PROMIS-

	SF-12 PCS: 46.7 ± 12.8 ; PROMIS-PF: 40.3 ± 12.3 ; SF-12 MCS: 52.1 ± 8.9 ; 6-Month Postoperative ; PROMIS-PF: 50.5 ± 11.7; PROMIS-PF: 47.0 ± 10.8 ; SF-12 PCS: 42.6 ± 15.1 ; SF-12 PCS: 35.6 ± 10.4 ; SF-12 PCS: 44.4 ± 12.9 ; SF-12 MCS: 52.2 ± 8.0 ; 1-Year Postoperative ; 12-Week Postoperative ; SF-12 MCS: 50.3 ± 14.2 ; PROMIS-PF: 40.8 ± 9.8					14.8 (Telemedicine followed by in-person)		PF at various follow-ups (preop, 12-week, six-month, and one-year follow-up).
**Crawford et al (2022)**						Change group (11) : 51.6 years (SD 23.0). - Overall mean age = 54.3 years ; No change group (292): 54.1 years (SD 17.3). ; Mean age across groups:	Success is inferred from the high accuracy (91.5%) of surgical plans via telemedicine.	
**Cui et al (2021)**	66% of patients seen in person after telemedicine triage had completed imaging, workup, and conservative management by the time of their clinic visit. ; 34% required additional tests or orders at the index visit.						40% “expedited” (<2 weeks) ; - Cost Savings: ; 15% “urgent” (<1 week) ; E-triage resulted in total patient cost savings of $793,835 over four years. ; - Efficiency: ; An average of $325 saved per patient ($266 in clinic fees and $59 in travel costs). ; 79.4% of patients offered appointments with spine surgeons were seen within six months. ; 21% of patients initially seen by spine surgeons underwent surgery within two years of their index visit. ; 34%	

							required additional tests or orders after their in-person visit. ; 1% same-day appointments. ; 57% of patients (5,842) were triaged to nonoperative providers, avoiding unnecessary appointments with spine surgeons. ; The E-triage process was completed within an average of 10 days from referral. ; 10,271 unique patients were reviewed through the E-triage system. ; Among patients seen in person: ; E-triage helped direct patients to appropriate care pathways, reducing unnecessary surgeon appointments and optimizing resource utilization.; Additional opportunity costs (e.g., time off work, child care) were not quantified but would further increase savings. ; 66% had completed imaging, workup, and conservative management by the time of their clinic visit. ; - Patient Triage Outcomes: ; - Diagnostic and Treatment Efficiency: ; 44% “next available” ; Patients who required surgical evaluation were assigned to appropriate appointment categories:	
**Farias et al (2022)**				Events involving post-operative		Mean age 54.8 years ± 16.2.	2. Weight Loss Success: ; Significant	High compliance with

				pain (n = 32) were rapidly detected and managed.			improvement in both functionality and quality of life was noted at 1 and 6 months post-surgery compared to the baseline. ; 3. Exercise Compliance: ; Among overweight patients (BMI > 25), 76% lost more than 3 kg with the support of remote nutritionists and specific exercises. ; 40% of events involving postoperative pain or wound issues were managed remotely, preventing unnecessary office or emergency visits.; 1. Improved Lumbar Spine Functionality and Quality of Life: ; 4. Avoidance of Unscheduled Care: ; 57% of patients were regularly exercising 6 months after surgery, contributing to overall recovery and success.	PROMs data input, with consistent engagement (94% at 1 month, 73% at 3 months).
**Goyal et al (2020)**								
**GrevenACM et al (2021)**					37% preferred telemedicine. ; 52% preferred in-person visits. ; 11% had no preference. ; Significant relationship between longer in-person travel times and telemedicine preference (P = 0.0007).	50–64 years: 34%. ; No significant correlation with telemedicine preference.; Mean age: 60 years (SD = 14). ; 18–49 years: 23%. ; 65+ years: 43%.	91% of patients were willing to use telemedicine again. ; 37% were comfortable proceeding with surgery based on telemedicine alone.; Measured in terms of satisfaction and willingness to use telemedicine again: ; 73% were comfortable proceeding with a minor procedure based on telemedicine alone.	

**GrevenACM et al (2022)**		Not significant: P = 0.317 ; Telemedicine group: 6.2 ± 2.6 ; Telemedicine group: -40.5% ± 54.3% ; Not significant: P = 0.811; Preoperative VAS: ; In-person group: -39.5% ± 66.6% ; Significant difference: P = 0.017 ; Postoperative Percentage Change in VAS: ; In-person group: -2.2 ± 3.7 ; Telemedicine group: -2.7 ± 3.1 ; Postoperative VAS Change: ; In-person group: 5.4 ± 2.8				In-person group: 61.4 ± 13.7 years ; Not significant: P = 0.485; Telemedicine group: 60.2 ± 14.3 years ; Mean Age:		
**Han et al (2021)**		Baseline VAS score: 8.2 ± 0.1 ; Post-implantation VAS score: 2.2 ± 0.1		Pain relief: 72.8% ± 1.7%	Preferred location for remote follow-ups: 79.7% prefer home ; Preferred remote follow-up methods: 46.9% prefer calls and local hospitals, 43.8% prefer video calls with remote programming ; Preferred remote programming method: Video calls and remote programming directly (43.8%)	Mean age: 58.6 ± 1.6 years		Daily life independency score: 5.2 ± 0.4; Post-implantation improvement score: 5.1 ± 0.3
**Hobson et al (2021)**					80% of patients reported they would choose telehealth again after their experience.	Mean age 55.1 years (SD 14.9)	91% successful connection on the first attempt for telehealth visits.	
**Jansen et al (2021)**						Mean = 59.3 ± 14.2 years	94% (preoperative plan did not change after in-person evaluation)	

**Leyendecker et al (2023)**		Neck pain: 4.1±0.6 to 2.3±0.5 (p<0.0001). ; Upper extremity pain: 2.6±0.6 to 1.1±0.3 (p=0.0012).; Significant reduction in pain levels:	Neck Disability Index (NDI) improved significantly postoperatively, from 18.6±2.5 to 9.1±2.5 (p=0.032).	Significant pain reduction postoperatively for both neck and upper extremities. Initial pain spike in the first three days, followed by a significant long-term decrease.	47.8% of patients preferred virtual follow-ups using the SPINEhealthie app, indicating openness to telemedicine.	Mean age of the cohort: 68.9±2.5 years.	The procedure was successful in reducing pain and improving mobility, with only one transient neurological deficit reported (resolved within a month). No permanent neurological deficits occurred.	Improvement in functional mobility suggested by stepping data: daily steps increased from 4,054±2,177 to 4,247±2,082 (though not statistically significant). ; Significant improvements in neck and upper extremity pain (VAS). ; Significant reduction in hospital stays and follow-ups for patients using telemedicine (p<0.0001).
**Leyendecker et al (2024)**	Higher virtual follow-up rates at 14 days (83.45%) and 90 days (76.52%) for SPINEhealthie.	Significant reduction in VAS neck/back and extremity pre-surgery values in SPINEhealthie (VAS neck/back = 4.88, VAS extremity = 4.78) compared to other groups (p < 0.05).	SPINEhealthie had significantly better ODI pre-surgery (18.08 ± 8.98) compared to 2020–2022 (23.01 ± 9.3) and 2018–2019 (22.56 ± 8.05), p < 0.001.	Significant pain regression observed at 2-week and 3-month timepoints across all groups, but no significant differences between them (p > 0.05).		SPINEhealthie: 58.4 (15.58) ; 2020–2022: 58.67 (16.35) ; The mean age across groups shows no significant difference: ; 2018–2019: 58.08 (15.47) ; p-value = 0.9.	reduced PROMs (VAS and ODI improvements), higher follow-up compliance rates, and better pain regression.	Clear improvements in VAS and ODI scores post-surgery across all timepoints, with SPINEhealthie showing the most compliance and moderate improvements.
**Li et al (2021)**			No difference at discharge (P>0.05). ; At the latest follow-up: Observation group: 22.9 ± 5.0, Control group: 30.1 ± 4.44 (P<0.05).	Pain regression was implied by the improved ODI and SF-36 scores in the observation group, indicating reduced disability and pain.		Overall mean age = 58.5 - not significant	- The observation group also had significantly better ODI and SF-36 scores.; Observation group: Pressure sores (5%), constipation (2.5%), joint stiffness (2.5%), muscle contracture (0%), foot drop (0%). ; - The observation group showed significantly fewer complications than the control group. ; Control	At the latest follow-up: Observation group: 75.8 ± 6.0, Control group: 63.2 ± 7.8 (P<0.05).; SF-36: No difference at discharge (P>0.05).

							group: Pressure sores (27.5%), constipation (15%), joint stiffness (17.5%), muscle contracture (10%), foot drop (10%).	
**LightseyHM4th et al (2022)**	Initial in-person visits were preferred for physical exams and rapport building; subsequent follow-ups (e.g., discussing imaging or postoperative care) were suitable for telemedicine.				Average preference for first-time in-person encounters: 7.7/10 (SD 3.5). ; Follow -up preferences were balanced (5.3/10, SD 3.8).; 81.9% preferred audiovisual telemedicine over audio-only.	Nonresponders: 59.5 years (SE 0.8, P = 0.07).; Responders: 61.8 years (SE 1.2)	High telemedicine satisfaction ratings suggest success in implementation but lower preference for initial consultations indicates limitations.	
**LowLYH et al (2024)**					Majority of patients preferred virtual multidisciplinary team discussions due to faster treatment, avoidance of long travel times, and convenience, particularly beneficial for those suffering from sciatica. ; Overwhelming majority were very satisfied with telemedicine consultations, with a few expressing limitations due to pain or physical issues like arm pain or ankle dorsiflexion difficulties		Success rates: 20% of patients underwent surgery, 14% had follow-up outpatient appointments, 7% were managed with physiotherapy, and 50% planned for spinal injections (outcomes and success rates in treatment pathways). ; no significant differences with respect to all motor scores, most sensory scores, and all special tests.	
**Master et al (2024)**		Leg pain: −4 (3 months), −3 (6 months) in the physical activity group.; Numerical Rating Scale (NRS) used instead: Back pain: −3 (3 months), −2.5 (6 months)	Usual care group at 3 months: −22 (Median) ; Physical activity group at 6 months: −24 (Median) ; Physical activity group at 3 months: −26 (Median) ; Usual care group at 6 months: −23 (Median)	NRS Back pain for usual care group at 3 months: −3 (Median) ; NRS Leg pain for usual care group at 3 months: −4.5 (Median) ; NRS Back pain for physical activity group at 3 months: −3	100% participants found the intervention useful; 88% preferred it for meaningful increases in physical activity.	Physical activity group: 71.5 years (Median) ; Usual care group: 61.0 years (Median) ; - Median age: 65.0 years ; - Mean age for all: 64.2 years (SD 13.2)	High retention (100%), adherence to intervention (88%), and achievement of step goals (75%).	ODI scores, NRS back pain, and NRS leg pain were improved in both groups over 3 and 6 months.

				(Median) ; NRS Leg pain for usual care group at 6 months: −5 (Median); NRS Leg pain for physical activity group at 3 months: −4 (Median) ; NRS Back pain for usual care group at 6 months: −3.5 (Median) ; NRS Leg pain for physical activity group at 6 months: −3 (Median) ; NRS Back pain for physical activity group at 6 months: −2.5 (Median)				
**Melian et al (2021)**					Teleconsultation preference was higher in city dwellers (58.9%) than in rural patients (47.6%). ; Postoperative visits: 87.5% preferred teleconsultation (126/144). ; Both males (54.5%) and females (56.1%) preferred teleconsultation (P<.001).; Checkups: 48.5% preferred teleconsultation (200/412). ; Initial consultations: 47.8% preferred teleconsultation (120/251).		Teleconsultation showed a high preference and satisfaction rate, indicating success in patient acceptance and usability, particularly during the COVID-19 lockdown.	
**Meron et al (2023)**					95% would recommend an initial telehealth appointment	39 (mean), 12 (SD)		
**Pennington et al (2022)**							Based on surgical yield data, in-person consultations yielded a higher success rate (35.5% overall surgical yield),	

							compared to telemedicine consultations, which had a lower success rate. Video consultations had an 18.6% surgical yield, and telephone consultations had 14.5%.	
**Perna et al (2021)**		Group B: ; Group A: ; T1 (15 days): 3.4 ± 2.1. ; T0: 7.6 ± 1.7. ; T2 (30 days): 1.1 ± 0.6. ; Statistical significance between groups: p = 0.003.; T1 (15 days): 1.8 ± 0.9. ; T2 (30 days): 2.1 ± 1.3. ; T0: 7.8 ± 1.4.	T1: 18.3 ± 2.7%. ; Group B: ; T0: 40.4 ± 3.6%. ; T1: 14.2 ± 2.4%. ; Group A: ; T2: 8.4 ± 2.1%. ; Statistical significance between groups: p = 0.001.; T0: 39.7 ± 4.4%. ; T2: 12.6 ± 2.4%.	Group A: 13.3 ± 2.4 days. ; Group B: 9.1 ± 1.6 days. ; Statistical significance: p = 0.0034.		Group A: 38.8±5.7 years ; Group B: 42.2±6.4 years	The treatment was more successful in Group B, with faster pain regression, better satisfaction rates, and fewer patients needing rescue drugs.	Group A had a higher rate of needing rescue medication compared to Group B (58.5% on Day 1 in Group A vs 13% in Group B).
**Prasse et al (2023)**		The average pain intensity measured by numerical rating scale (NRS) was 4.7 (SD ± 2.3).				60		
**Rappard et al (2023)**		87% (65/75) had NDI ≥20. ; 95% CI [29, 51]). ; - NDI Outcomes: ; - Combined MCID Results: ; 95% CI [43, 66]). ; MCID for NDI: Achieved in 55% (41/75; 85% (64/75) had VAS ≥5. ; VAS and NDI Together: Achieved in 40% (30/75; VAS or NDI: Achieved in 71% (53/75; Presenting NDI: 46 (95% CI [41, 51]); 95% CI [46, 69]). ; Post-Treatment NDI: 30 (95% CI [24, 36]). ; Post-Treatment VAS: 4 (95% CI [3,	- ODI Outcomes: ; - Combined MCID Results: ; 95% CI [60, 80]).; Post-Treatment ODI: 35 (95% CI [30, 40]). ; VAS or ODI: Achieved in 70% (56/80; 95% CI [37, 58]). ; 95% CI [49, 71]). ; MCID for ODI: Achieved in 60% (48/80; Presenting ODI: 52 (95% CI [48, 56]); 95% (76/80) had ODI ≥20. ; VAS and ODI Together: Achieved in 48% (38/80	87% (65/75) had NDI ≥20. ; - VAS Outcomes (Pain Regression): ; 85% (64/75) had VAS ≥5. ; Pain Regression (VAS): ; Overall: 16 points reduction on average.; Presenting NDI: 46 (95% CI [41, 51]); - NDI Outcomes (Pain Regression): ; Post-Treatment VAS: 4 (95% CI [3, 5]). ; Post-Treatment NDI: 30 (95% CI [24, 36]). ; Pain Regression (NDI): ; Overall: 3 points reduction on average. ; Presenting VAS: 7 (95% CI [7, 8])		Range: 18–80 years. ; 89% were aged 60 or below.; Mean : 42 years.	Cervicothoracic patients achieved MCID for VAS or NDI in 71% of cases. ; Low back pain patients showed a reduction in VAS from 7 to 5 and ODI from 52 to 35.; - High MCID Achievement Rates: ; Low back pain patients achieved MCID for VAS or ODI in 70% of cases. ; - Significant Pain and Disability Reduction: ; Cervicothoracic patients experienced a reduction in VAS from 7 to 4 and NDI from 46 to 30.	Visual Analog Scale (VAS) ; Neck Disability Index (NDI) ; Oswestry Disability Index (ODI) ; Minimal Clinically Important Difference (MCID)

		5]). ; MCID for VAS: Achieved in 57% (43/75; - VAS Outcomes: ; 95% CI [60, 81]).; Presenting VAS: 7 (95% CI [7, 8])						
**Satin et al (2022)**					45% prefer telemedicine for future visits	Median age 60-69 years		
**Shafi et al (2020)**					86.9% of patients would recommend telemedicine to a friend. Only one patient (1.2%) stated they would not recommend it, while 10.7% were uncertain.	Majority of respondents were over 60 years old (63.1%). ; No significant differences in satisfaction or effectiveness scores were observed based on age (p > 0.05).	High satisfaction scores and recommendation rates indicate success. However, technical issues were noted by 11.9% of patients, and the lack of physical examination was a recurring critique.	
**Shah et al (2024)**		- MCID Achievement for NPRS: ; - Post-treatment NPRS Score: ; Telerehabilitation Group: 1±1.6 (95% CI: 0.8–1.1). ; Percentage achieving MCID of 2: ; - Baseline NPRS Score: ; Control: 90% ; Mean: 7±2 (95% CI: 6.8–7.1) in both groups. ; No significant difference (p = 1.00). ; Mean Difference: 1 point, p < 0.0001 (significant reduction in Telerehabilitation Group). ; Tele-rehabilitation: 96% (p = 0.0007). ; NPRS 8: Telerehabilitation 97.5% vs. Control 84.5% (mean difference: 13%, p < 0.0001).; Con	- Baseline ODI/NDI Score: ; Control: 47.2±16.7 (95% CI: 45.6–48.7). ; - Percentage achieving MCID of 30% for ODI/NDI in pre-treatment categories: ; Telerehabilitation Group: 17±15 (95% CI: 15.5–18.4). ; - MCID Achievement for ODI/NDI: ; Severe Disability: Telerehabilitation 85.5% vs. Control 73.5% (mean difference: 12%, p = 0.002). ; Mean: ; Telerehabilitation: 46±16 (95% CI: 44.4–47.5). ; - Post-treatment ODI/NDI Score: ; Minimal Disability: Telerehabilitation 79% vs. Control 50% (mean	Pain regression: 6.7 - 1.0 = 5.7 ; - Control group (All patients): ; Pain regression: 7 - 2 = 5 ; Pre-treatment NPRS: 6.7 ± 1.9 ; Telerehabilitation group shows greater pain regression (mean difference = 1, p < 0.0001) compared to the control group. ; Pain regression by location (Telerehabilitation group): ; Pre-treatment NPRS: 6.7 ± 1.8 ; Pre-treatment NPRS: 7.0 ± 2.0 ; The telerehabilitation group had significant reductions in pain across all measured locations except for certain subgroups where		<30 years: 51 (12%) in both groups ; >60 years: 322 (75%) in both groups; 31–60 years: 55 (13%) in both groups	- Low Back Pain (LBP): ; No significant difference in post-treatment NPRS and ODI/NDI scores. ; - Neck and Upper Back Pain (NP+UBP): ; Significant improvement in post-treatment NPRS and ODI/NDI scores for Telerehabilitation. ; MCID achievement for ODI/NDI (10): Telerehabilitation 85% vs. Control 75.5% (p = 0.002). ; - Neck Pain (NP): ; Significant reduction in post-treatment ODI/NDI scores for Telerehabilitation (mean difference: 9.9, p = 0.007).; MCID achievement for NPRS (2): Telerehabilitation 96% vs. Control 89% (p = 0.0004).	ODI/NDI, NPRS (Numerical Pain Rating Scale) scores.

		trol Group: 2±2 (95% CI: 1.8–2.1). ; NPRS 4–7: Telerehabilitation 96% vs. Control 88.5% (mean difference: 7.5%, p = 0.001). ; Percentage achieving MCID of 30% for NPRS in pre-treatment categories:	difference: 29%, p = 0.03). ; Mean Difference: 5.8 points, p < 0.0001 (significant reduction in Telerehabilitation Group). ; Control: 77.5% ; Crippled/Bed-Bound: Telerehabilitation 86.5% vs. Control 73.5% (mean difference: 13%, p = 0.04).; Telerehabilitation: 85% (p = 0.005). ; Control Group: 22.8±17.7 (95% CI: 21.1–24.4). ; Percentage achieving MCID of 10: ; No significant difference (p = 0.13).	differences were non-significant.; Post-treatment NPRS: 2 ± 2 ; Post-treatment NPRS: 1.0 ± 1.0 ; Pre- and Post-Treatment NPRS Scores: ; Pre-treatment NPRS: 7 ± 2 ; Pain regression: 7 - 1 = 6 ; Post-treatment NPRS: 1 ± 1.6 ; Pain regression: 7.0 - 1.0 = 6.0 ; Post-treatment NPRS: 1.2 ± 1.6 ; 3. Neck and upper back pain (NP + UBP): ; Post-treatment NPRS: 1.0 ± 1.7 ; Pain regression: 6.7 - 1.2 = 5.5 ; 2. Neck pain (NP): ; - Telerehabilitation group (All patients): ; Comparative Pain Regression: ; 1. Low back pain (LBP):				
**Sharma et al (2021)**					Higher satisfaction with virtual visits for appointment availability, provider care, and recommendation likelihood.		Virtual consultations maintained or slightly improved satisfaction and diagnostic consistency compared to in-person visits.	
**Sultan et al (2020)**						General pediatric orthopaedic patients: Mean age = 12 ± 4.7 years.; PSD patients: Mean age = 15 ± 3.7 years.	Telemedicine visits resulted in favorable satisfaction and significantly reduced wait times for PSD patients.	
**Tian et al (2020)**	N.A					Mean = 52.5 years (range: 23–71 years)	All operations were performed as planned; no intraoperative adverse events were observed. The deviation between the	

							planned and actual positions of the pedicle screws was 0.76 ± 0.49 mm, which is within an acceptable range. Pedicle screw placement accuracy was high, with 95.2% being grade A and 4.8% being grade B according to the Gertzbein-Robbins criteria. The acceptable rate of screw placement was 100%.	
**Totala et al (2024)**	Approximately 22.22% of patients required a physical visit after the first teleconsultation for detailed evaluations. This increased to 29.41% for the second visit and 50% for the third visit.				Patients living more than 100 km from the hospital were predominant in the study (60.71%), indicating that telemedicine was preferred for reducing travel. The total travel distance saved was 12,322 km, reducing CO2 emissions by 1,763.2 kg, suggesting environmental and logistical benefits.	mean age of the study group was 40.42 years (range: 1–81 years). Most patients (56%) were between 21 and 50 years old.	Overall success rate of teleconsultations was 82.87%. Success was significantly associated with socioeconomic and educational statuses (e.g., upper class: 100%, lower class: 44%, $p < 0.05$).	
**Woznica et al (2023)**	Significance: N/A; No separate PROMs values or P-values reported for this subgroup. ; Only 16.7% (n=6) required follow-up physician visits.			NPRS final mean: 2.1 ± 1.9 (P=<0.0001). ; NPRS initial mean: 5.6 ± 1.8. ; Significance: Highly significant; No patients reported worsening of pain. ; NPRS at 30 days: 3.2 ± 1.6. ; Improvement in 91.6% (n=33) of patients. ; Significance: Highly significant (P<0.0001) - NPRS improved in 91.6% (n=33) of patients (P=<0.0001).		Mean age: 53.5 years (±15.3). ; Significance: N/A; No direct significance stated.	Significance: Highly significant; Improvements in pain (91.6%, P=<0.0001) and HRQoL (PROMIS-MH and PROMIS-PH).	NPRS improvement: 91.6% (n=33) (P=<0.0001). ; PROMIS-MH: Improvement of 3.4 points (P=0.0293). ; Significance: Statistically significant; PROMIS-PH: Improvement of 4.7 points (P=0.0122).

**Ye et al (2022)**	P = 0.002, statistically significant).; Delay observed in telemedicine group (mean of 44 days to surgery vs. 33 days in in-person group					Overall: 54.9 years (95% CI: 52.5–57.3). ; No significant difference in age distribution (P = 0.268).; Mean age: ; In-person: 55.8 years (95% CI: 53.0–58.7). ; Telemedicine: 52.7 years (95% CI: 48.4–57.1).	No significant difference (P = 0.673).; Telemedicine: 79.5%. ; Surgical plan success (no change needed): ; In-person: 82.6%.	
**Ye et al (2024)**						In-person: 55.0 ± 16.7 years (95% CI, 54.5–55.5) ; Telemedicine: 55.6 ± 15.5 years (95% CI, 55.2–56.1) ; P-value = 0.101 (not significant) - Overall mean age = 55.3 years	Sex: More female patients had telemedicine visits (57.1%) compared to in-person appointments (53.8%) with a P-value of 0.003, which is significant. ; Race: Significant differences were noted between telemedicine and in-person visits in terms of racial distribution, with a P-value < 0.001. ; Area Deprivation Index (ADI): Patients with higher ADI were more likely to miss in-person appointments, and those with low or medium ADI were more likely to miss telemedicine visits (all P-values < 0.001). ; Socioeconomic status: The relationship between ADI and missed visits was highly significant (P < 0.001), with a clear trend that higher ADI was associated with more misse in-person visits.; Missed Visits: Telemedicine visits had a significantly lower missed visit rate	

							(24.7%) compared to in-person visits (51.3%), with a P-value < 0.001, indicating telemedicine visits were more likely to be attended.	
**Zhu et al (2021)**						Mean patient age was 12.4 ± 1.5 years (range: 10–15 years)	Significant improvements in compliance: Quality compliance rose from 49.1% to 80.5%, and quantity compliance from 52.3% to 70.3% over 6 months (P<0.05).	

Supplementary Table 2 – Key clinical and functional outcomes of the analysed studies
